# Implicit neural sensitivity for negatively valued social and non-social visual scenes in young adults exposed to childhood adversity

**DOI:** 10.1017/S0033291725000029

**Published:** 2025-02-11

**Authors:** Zhiling Qiao, Stephanie Van der Donck, Victor Mazereel, Lise Jennen, Celine Samaey, Davy Vancampfort, Ruud van Winkel, Bart Boets

**Affiliations:** 1 Department of Neurosciences, Research Group Psychiatry, Center for Clinical Psychiatry, KU Leuven, Leuven, Belgium; 2 Leuven Brain Institute (LBI), KU Leuven, Leuven, Belgium; 3 Department of Neurosciences, Research Group Psychiatry, Center for Developmental Psychiatry, KU Leuven, Leuven, Belgium; 4 Department of Rehabilitation Sciences, KU Leuven, Leuven, Belgium; 5 University Psychiatric Center (UPC), KU Leuven, Leuven, Belgium; 6 Child and Youth Institute, KU Leuven, Leuven, Belgium

**Keywords:** childhood adversity, electroencephalography, emotion processing, neglect, psychopathology, threat

## Abstract

**Background:**

Based on facial expression experiments, childhood adversity may be associated with threat-related information processing bias. Yet, it is unclear whether this generalizes to other threat-related stimuli, such as social and non-social visual scenes.

**Methods:**

We combined fast periodic visual stimulation with frequency-tagging electroencephalography (EEG) and eye-tracking to assess automatic and implicit neural discrimination, neural salience and preferential looking towards negative versus neutral social and non-social visual scenes in young adults aged 16–24 years (51 with childhood adversity and psychiatric symptoms and 43 controls).

**Results:**

Controls showed enhanced negative-neutral neural discrimination within a social versus non-social context. However, this facilitating effect of social content was absent in those with adversity, suggesting a selective alteration in social threat processing. Moreover, individual differences in adversity severity, and more specifically threat experiences (but not neglect experiences), were associated with decreased neural discrimination of negative versus neutral social scenes, corresponding to similar findings in facial expression processing, indicating the robustness of adversity-related deficits in threat-safety discrimination across social visual stimuli.

**Conclusions:**

The adversity-related decreased threat-safety discrimination might impact individuals’ perception of social cues in daily life and relate to poor social functioning and future development of psychopathology.

## Introduction

Childhood adversity is the single most important and shared risk factor for internalizing, externalizing, and psychotic disorders (Baldwin et al., 2023). Approaches to understanding these associations have emphasized a threat-related information processing bias (Lecei & van Winkel, 2020; Mclaughlin & Lambert, 2017). These include facilitated perceptual sensitivity, e.g. physically abused children were able to identify angry faces with less sensory input (Pollak & Sinha, 2002), and attentional bias, e.g. victimized children showed rapid attention engagement towards and delayed disengagement from angry faces (Pollak & Tolley-Schell, 2003). This atypical threat-related processing bias, however, has mainly been observed using images of facial expressions presented in isolation, that is context-free faces, which may not reflect emotion processing within more complex real-world environments. Thus, it is unclear whether the broadly observed adversity-related alterations in threat processing can also be demonstrated in the processing of more naturalistic emotional scenes.

Social content is a unique and highly relevant dimension that affects emotion processing (Löw, Bradley, & Lang, 2013). Prioritized processing of social over non-social information has been well demonstrated in human beings across the lifespan (Lee & Green, 2016). New-borns preferentially track moving stimuli of a face pattern as compared to other patterns (Goren, Sarty, & Wu, 1975); children exhibit larger neural responses and preferential looking towards faces versus houses (Vettori et al., 2020); and adults saccade rapidly towards faces even when they appear next to images of non-social stimuli such as vehicles (Crouzet, Kirchner, & Thorpe, 2010). Social cues prompt the neural perceptual processing of emotional images, such as enhanced neural activation towards images that include people versus those that do not (Löw et al., 2013). Alterations in the emotional processing of this social dimension have been related to psychopathology (Pinkham et al., 2014; Zivotofsky, Oron, Hibsher-Jacobson, Weintraub, & Strous, 2008). Whereas healthy controls showed higher arousal responses to social versus non-social unpleasant pictures, individuals with schizophrenia did not show this distinction, and their lower arousal responses to unpleasant social (but not non-social) pictures uniquely related to their negative symptoms (Bodapati & Herbener, 2014). This raises another underexplored question: does childhood adversity increase the vulnerability for psychopathology by specifically altering the emotional processing of social cues?

The current study investigates the processing of complex social versus non-social visual scenes and the modulating effect of childhood adversity, by applying fast periodic visual stimulation in combination with frequency-tagging electroencephalography (EEG). This approach relies on the principle that the human brain synchronizes its activity to the periodic rate of a flickering stimulus (Adrian & Matthews, 1934). As it elicits brain responses tagged exactly to the presentation rate (Norcia, Gregory Appelbaum, Ales, Cottereau, & Rossion, 2015), it allows us to objectively mark and quantify the automatic neural processes without explicit task demands, even when stimuli are presented fast and simultaneously. Firstly, by administering an oddball paradigm, we quantified the neural discrimination of quickly presented negative versus neutral visual scenes, with either social or non-social content in different trials. Secondly, we administered a multi-input paradigm in combination with EEG and eye-tracking to investigate the neural salience and overt attentional orienting towards simultaneously presented negative versus neutral visual scenes, again with social or non-social content across different trials.

In addition, in line with the dimensional framework of childhood adversity, we distinguished between experiences of threat (that is domestic violence, peer and sibling victimization, and physical, emotional, and sexual abuse) and experiences of deprivation (that is physical and emotional neglect), as they may differentially impact on emotional processing (McLaughlin, Sheridan, & Lambert, 2014). Indeed, as demonstrated with regard to facial expression processing, threat but not deprivation experiences elicit a threat-related information processing bias (Qiao et al., 2024). Thus far, no study has investigated the potentially distinct impact of these two adversity dimensions on the processing of complex scenes. Furthermore, given the link between childhood adversity and psychopathology (Baldwin et al., 2023) and as alterations in emotion processing are also observed in psychiatric disorders (Catalan, Artaza, Bustamante, & Orgaz, 2016; Cotter et al., 2018; Pena-garijo et al., 2023), here, we study victims of childhood adversity who already present (sub-)clinical symptoms.

More specifically, we investigated an adversity group, with individuals exposed to childhood adversity presenting with (sub-)clinical symptoms of depression, anxiety, and/or psychosis, aged 16–24 years (that is a period during which psychopathology most often emerges; Solmi et al., 2022), and a healthy control group. In previous work on this population (Qiao et al., 2024), we reported reduced angry-neutral face discrimination with increasing threat exposure, potentially resulting from a negative perception of neutral faces. Therefore, we hypothesize that this may extend to decreased neural discrimination of negative versus neutral scenes, particularly those with social content. Furthermore, we hypothesize that while both groups may avoid looking at negative scenes and prefer looking toward neutral scenes, the adversity group may show higher neural salience toward the negative scenes as compared to the control group in the social context.

## Methods

### Participants

Based on exposure to childhood adversity and the presence of psychiatric symptomatology, two groups of participants aged 16–24 years old were delineated during the screening session: (i) an adversity group (n = 52), scoring above the threshold of childhood adversity and presenting at least two symptom dimensions (depressive, anxiety and psychotic symptoms) (Van Nierop et al., 2015), and (ii) a control group (n = 61) with participants scoring below all of the thresholds. During the testing session, adversity and symptomatology were measured again, to assess individual differences in general adversity exposure, threat and neglect experiences of adversity, and current symptoms (see full details in Supplementary Methods).

### Stimuli and procedure

**Figure 1.**
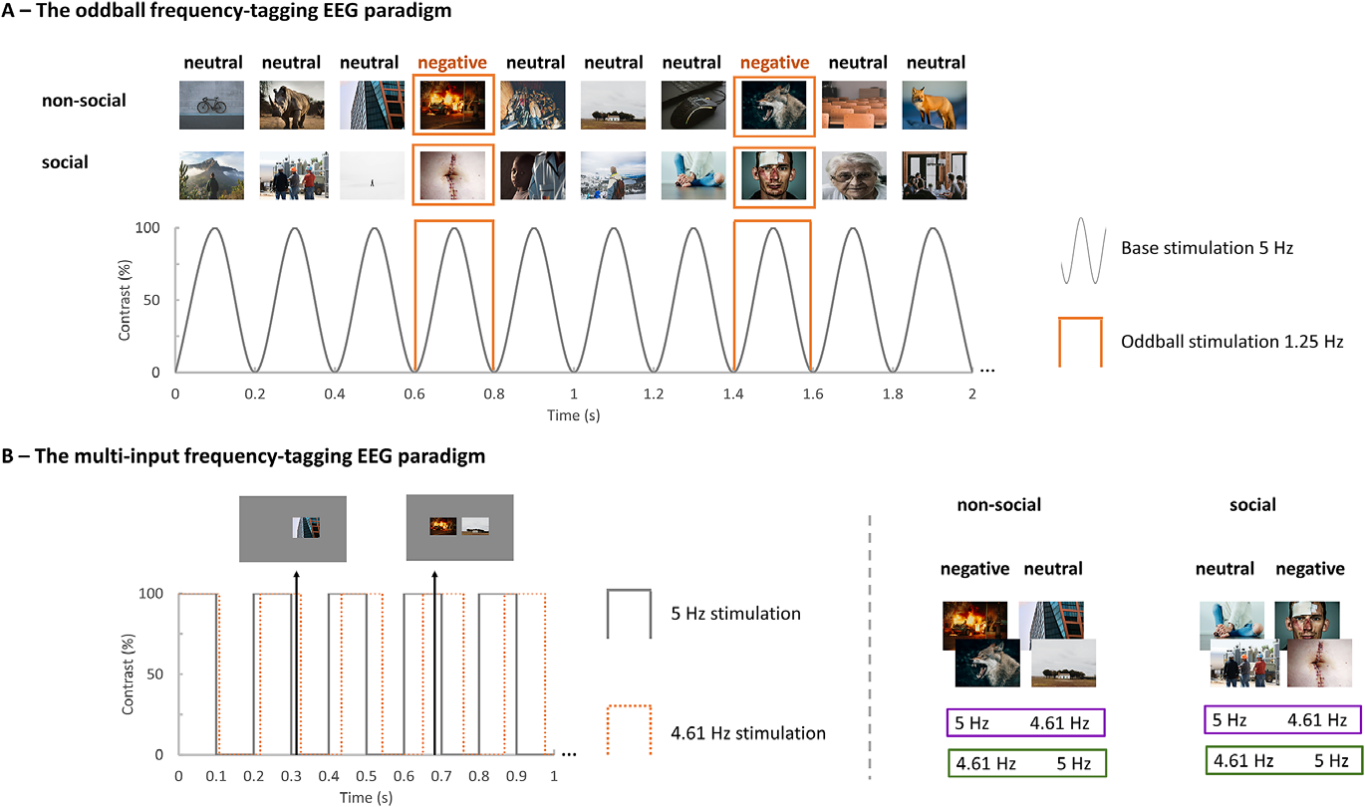
The fast periodic visual stimulation oddball and multi-input paradigms. (a) Illustration of a stimulation sequence for the oddball paradigm, with neutral scenes presented at a 5 Hz base rate, periodically interleaved with a negative scene every fourth image (1.25 Hz oddball rate). Two categories of sequences, that is scenes with either social or non-social content, were administered in distinct trials. (b) Illustration of a stimulation sequence for the multi-input paradigm, with negative scenes presented at 5 Hz in the left visual field and neutral faces presented at 4.61 Hz in the right visual field. The first black arrow indicates that the negative scene was presented at 0% and the neutral scene was presented at 100% contrast. The second black arrow indicates that both the negative and neutral scenes were presented at 100%. Exemplary images (from the free image database site https://unsplash.com/) are presented here since the inclusion of images from the NAPS in a scientific publication is not allowed.

Stimuli were selected from the Nencki Affective Picture System (Marchewka, Żurawski, Jednoróg, & Grabowska, 2014) and consisted of four categories of 25 images each, displaying either a negative social scene (e.g. mutilated body and sad woman), a neutral social scene (e.g. builder and garbage collector), a negative non-social scene (e.g. dead cat and fire) and a neutral non-social scene (e.g. cat and window). For the oddball frequency-tagging EEG paradigm (Figure 1a), a series of base stimuli (that is neutral scenes) were displayed at 5 Hz, periodically interleaved with a negative scene every fourth image (that is oddball stimuli displayed at 5 Hz/4 = 1.25 Hz). The multi-input paradigm (Figure 1b) consisted of two simultaneously presented streams of negative and neutral scenes, each labeled at a distinct presentation rate (that is 4.61 Hz versus 5 Hz, or vice versa) and displayed at either the left or right visual field. Scenes with social or non-social content were displayed in different trials in both paradigms, with the order of the trials randomized across participants (see full details in Supplementary Methods).

### Data analysis

**EEG data** was preprocessed using Letswave 6 (https://www.letswave.org/) and MATLAB 2021 (see Supplementary Methods).

After preprocessing, the data were transformed from the time domain to the frequency domain using a fast Fourier transformation. The frequency data contain the neural responses tagged to the stimulation frequencies and their harmonics (that is integer multiples). To visualize the responses, we calculated the signal-to-noise ratio (SNR) by dividing the amplitude value of the stimulation frequencies and their harmonics by the average amplitude of the 20 surrounding frequency bins (Dzhelyova, Jacques, & Rossion, 2017). To quantify the neural sensitivity for (non-)social scenes, we calculated the baseline-subtracted amplitude at the stimulation frequencies and their harmonics by subtracting the average amplitude level of the 20 surrounding bins from the amplitude of the target frequency bin (Liu-Shuang, Norcia, & Rossion, 2014). Harmonics were included if the Z-score (that is calculated based on the mean and standard deviation of these 20 surrounding frequency bins) for two consecutive harmonics was above 1.64 (*p* < 0.05) in all regions of interest (ROIs) for both groups in the social and non-social context. Consequently, oddball discrimination responses were quantified as the summed responses of the first five harmonics without the harmonic corresponding to the 5 Hz base rate frequency (that is until 5F/4 = 7.5 Hz). For the multi-input paradigm, neural responses were quantified as the summed responses of the first four harmonics for both stimulation frequencies (that is until 18.44 Hz and 20 Hz for 4.61 Hz and 5 Hz, respectively).

Based on previous studies (Qiao et al., 2022; Van der Donck et al., 2019) and supported by visual inspection of the topographical maps, we defined the following ROIs for the oddball paradigm: left occipito-temporal (LOT; P7, P9, PO7), medial occipital (MO; Iz, Oz, O1, O2) and right occipito-temporal (ROT; P8, P10, PO8). The same MO region was selected for the multi-input paradigm.

**Eye-tracking data** was analyzed using a series of custom-built scripts in Matlab (Supplementary Methods). Two areas of interest (AOI) were defined as the rectangular areas where the two images of scenes were presented. An additional ‘outside AOI’ was defined to label all the fixation points that were not attributed to the two AOIs. Proportional looking times for each AOI were then quantified as the relative duration of all fixation points allocated to that AOI using a probability weighting approach while taking the subject-specific data quality into account (Vettori et al., 2020).

### Statistical analysis

We performed linear mixed models (LMM) using the *R*-package *afex*, version 1.2.1 (Singmann, Bolker, Westfall, Aust, & Ben- Shachar, 2020), and *nlme*, version 3.1–163 (Pinheiro, Bates, & Team, 2023), in *R*-statistical software, version 4.1.3 (R Core Team., 2022). Tukey-corrected post-hoc t-tests were used to compare means using the *R*-package *emmeans*, version 1.7.3 (Lenth, Singmann, Love, Buerkner, & Herve, 2022).

For both paradigms, a first model was constructed to compare the control and adversity group, followed by a second model to investigate the impact of adversity exposure (as a continuous measure) while controlling for symptoms within the adversity group, and a third model to pinpoint the unique effect of threat and neglect experiences. Thus, for the neural discriminative responses measured in the oddball paradigm, we constructed the following three models: (1) Model_1_, y ~ age + sex + Group * Content * ROI + (1|subject), with Group [adversity versus control] as the between-subjects factor, and Content [social versus non-social] and ROI [LOT, MO, ROT] as the within-subjects factors; (2) Model_2_, y ~ age + sex + Childhood adversity * Content + Depression * Content + Anxiety * Content + Psychosis * Content + (1|subject), with all measures as continuous predictors within the CA group; (3) Model_3_, y ~ age + sex + Threat * Content + Neglect * Content + Depression * Content + Anxiety * Content + Psychosis * Content + (1|subject), to further investigate the effects of threat and neglect experiences as continuous measures within the adversity group. Three similar models were constructed for the neural salience and looking patterns measured in the multi-input paradigm, with Valence [negative versus neutral] and Content [social versus non-social] as within-subject effects. During analyses, 6 out of 546 data points (that is one data point for each ROI under each condition per participant) for the oddball paradigm and 20 out of 728 data points (EEG data) and 1 out of 552 data points (eye-tracking data) (that is one data point for each valence and content under each presentation rate per participant) for the multi-input paradigm were detected as outliers using the median absolute deviation and were removed. The continuous adversity and symptomatology measures were standardized (*M* = 0, *SD* = 1) before being entered into the models. Exploratory analyses were conducted to investigate the effect of adversity and symptoms while not controlling for each other (Supplementary Methods and Results).

## Results

### Demographic information

As the current study is part of a large project and was initiated later, EEG data of the two paradigms was only collected in 43 controls and 51 participants with adversity. Three participants with adversity were excluded due to missing data on psychiatric symptomatology, resulting in a final sample of 48 participants with adversity. The eye-tracking data of the multi-input paradigm was collected for 34 controls and 47 participants with adversity. The same two participants exposed to adversity with missing symptom measures were excluded. Additionally, seven participants (four controls) were excluded as their data did not meet the accuracy threshold during calibration validation, and three participants (two controls) were excluded due to wrongly recorded data, resulting in a final sample of 28 controls and 41 participants with adversity. Group demographic characteristics are reported in Table 1.

**Table 1.** Demographic information of the final sample that included in each paradigm

		The oddball and multi-input paradigms – EEG			The multi-input paradigm – eye-tracking		
Characteristics		Controls	Adversity	*p*-value	Controls	Adversity	*p* value
Sex		30 F/13 M	31 F/17 M	.599	20 F/8 M	25 F/16 M	.371
Age, Range (Mean ± SD)		17–24 (21.3 ± 1.9)	16–24 (20.0 ± 2.2)	.003	17–24 (21.5 ± 2.1)	16–24 (20.2 ± 2.2)	.017
Childhood adversity, Range (Mean ± SD)							
Screening	Adversity	0–0.21 (0.04 ± 0.05)	0.10–1.73 (0.58 ± 0.43)	< .001	0–0.18 (0.03 ± 0.05)	0.12–1.73 (0.57 ± 0.43)	< .001
	Threat	0–0.30 (0.05 ± 0.08)	0–1.98 (0.55 ± 0.41)	< .001	0–0.26 (0.04 ± 0.06)	0–1.98 (0.53 ± 0.41)	< .001
	Neglect	0–0.40 (0.01 ± 0.06)	0–2.75 (0.66 ± 0.72)	< .001	0–0.40 (0.01 ± 0.08)	0–2.75 (0.70 ± 0.74)	< .001
Testing	Adversity	0–0.30 (0.03 ± 0.05)	0–1.63 (0.52 ± 0.43)	< .001	0–0.14 (0.03 ± 0.04)	0–1.41 (0.49 ± 0.41)	< .001
	Threat	0–0.41 (0.04 ± 0.07)	0–1.48 (0.47 ± 0.39)	< .001	0–0.13 (0.03 ± 0.04)	0–1.25 (0.44 ± 0.36)	< .001
	Neglect	0–0.50 (0.02 ± 0.09)	0–2.60 (0.64 ± 0.78)	< .001	0–0.50 (0.03 ± 0.11)	0–2.60 (0.61 ± 0.75)	< .001
Symptoms, Range (Mean ± SD)							
Screening	Depression	0–8 (3.0 ± 2.5)	11–45 (20.7 ± 8.6)	< .001	0–8 (2.9 ± 2.4)	11–45 (20.3 ± 7.9)	< .001
	Anxiety	21–39 (31.8 ± 4.8)	41–73 (55.4 ± 8.2)	< .001	21–39 (32.4 ± 4.7)	41–72 (55.0 ± 7.7)	< .001
	Psychosis	0–4 (0.7 ± 1.0)	0–11 (4.3 ± 2.5)	< .001	0–4 (0.8 ± 0.9)	0–11 (4.3 ± 2.4)	< .001
Testing	Depression	0–11 (2.9 ± 3.0)	1–42 (19.4 ± 10.4)	< .001	0–8 (2.4 ± 2.6)	1–41 (18.8 ± 10.0)	< .001
	Anxiety	20–42 (30.7 ± 5.3)	29–74 (53.2 ± 10.3)	< .001	20–37 (30.5 ± 4.5)	29–71 (52.3 ± 9.8)	< .001
	Psychosis	0–6 (1.1 ± 1.5)	0–14 (5.2 ± 3.4)	< .001	0–4 (1.1 ± 1.3)	0–14 (5.2 ± 3.4)	< .001

Legend: For each group, the range (mean ± SD) of age and childhood adversity and psychiatric symptoms measured during the screening and testing session is reported. Regarding childhood adversity, a continuous score was created by averaging the mean frequency (that is the mean score across the related items for each adversity category) of all the seven categories of childhood adversity (peer and sibling victimization/bullying, physical abuse, physical neglect, emotional abuse, emotional neglect, sexual abuse and domestic violence), measured using a modified version of the Juvenile Victimization Questionnaire 2nd revision (Adult Retrospective Form; JVQ-R2) and 5 questions of the Emotional Neglect subscale of the Childhood Trauma Questionnaire (CTQ). Neglect exposure was indicated by the average score of physical and emotional neglect, while threat exposure was indicated by the average score of the other five categories. 29 participants scored above zero on the neglect dimension and 44 participants scores above zero on the threat dimension. Depressive, anxiety and psychotic symptoms were measured using the Beck Depression Inventory (BDI-II), the trait scale of the State Trait Anxiety Inventory and the Prodromal Questionnaire-16 (PQ-16), separately. The groups were compared using a two- sample t-test for continuous measures and a chi-squared test for sex.

### Discrimination of negative versus neutral scenes with social and non-social content in the oddball paradigm

**Figure 2.**
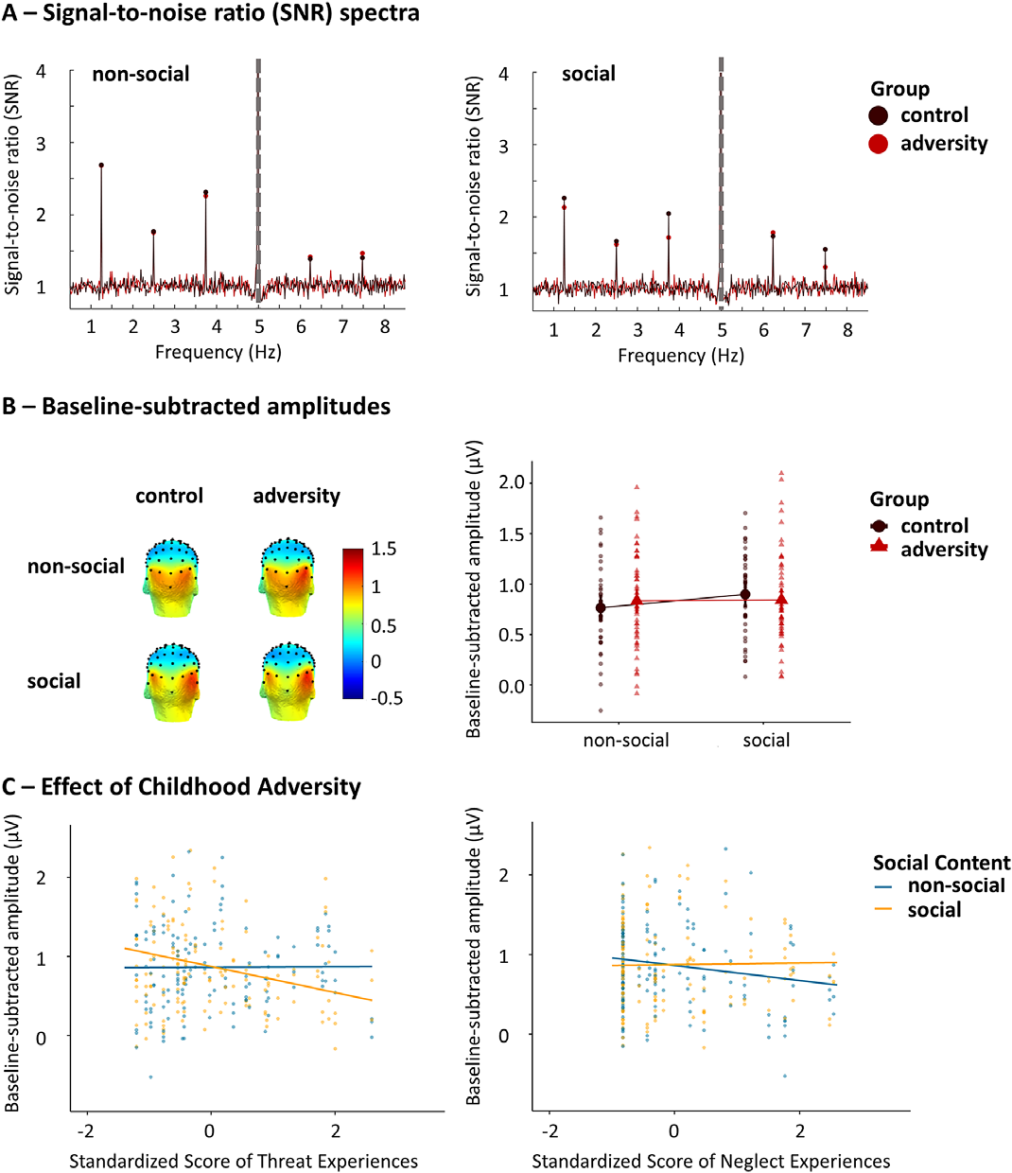
(a) SNR EEG spectra of the neural discrimination responses averaged across the three ROIs for each group in the social and non-social content streams. The significant first five harmonics are displayed. The dashed line indicates the 5 Hz base rate response. (b) Left: Scalp distributions of the EEG signal based on the baseline-subtracted amplitudes in μV for each content condition and each group. Across the two groups, the neural responses in the social but not the non-social context were right-lateralized. Right: Baseline-subtracted amplitudes averaged across the five harmonics and three ROIs for each content condition and each group. While controls showed larger neural discrimination responses for social negative scenes versus non-social negative scenes, those did not differ in the adversity group. (c) Left: Threat experiences were associated with decreased negative-neutral scene discrimination for social versus nonsocial content. Right: There was also a tendency (*p* = .05) that neglect experiences were associated with decreased negative-neutral scene discrimination for non-social versus social content. Standardized scores were used.

Negative scenes elicited robust discrimination responses at the oddball frequency and harmonics for both groups in both the social and non-social stimulation streams (Figure 2a). The first Model_1_, contrasting the control and adversity group, revealed no main effect of group (*F*_(1,87)_ = 0.01, *p* = .947), a main effect of content (*F*_(1,440)_ = 5.35, *p* = .021), and a significant group by content interaction effect (*F*_(1,440)_ = 4.03, *p* = .045), which was driven by larger neural discrimination responses for social relative to non-social negative scenes in the control group (*t*_(440) nonsocial-social_ = −2.97, *p* = .016), but not in the adversity group (*t*_(439) nonsocial-social_ = −0.22, *p* = .996) (Figure 2b). Thus, while the social content facilitated the discrimination of negative scenes from neutral scenes in controls, this social facilitatory effect was not observed in participants with adversity. There was also a main effect of ROI (*F*_(2,439)_ = 12.75, *p* < .001) and a significant ROI by content interaction effect (*F*_(2,439)_ = 14.07, *p* < .001), indicating right lateralized neural responses in the social context (*t*_(439) social LOT-ROT_ = −4.05; *t*_(439) social MO-ROT_ = −6.03, both *p* < .001) and larger responses at the MO than LOT in the non-social context (*t*_(439) nonsocial LOT-MO_ = −3.82, *p* = .002). Additionally, relative to non-social context, social context yielded larger responses in the LOT and ROT (*t*_(439) LOT nonsocial-social_ = −2.89, *p* = .046; *t*_(440) ROT nonsocial-social_ = −4.07, *p* = .001) regions, but smaller responses in the MO (*t*_(440) MO nonsocial-social_ = 2.94, *p* = .041) region. No other significant effects were observed (all *p* > .16).

Model_2_, investigating the response pattern in relation to individual differences in adverse experiences within the adversity group while controlling for symptomatology, did not reveal any significant effect (all *p* > .09). Model_3_, disentangling the effect of threat and neglect experiences, showed a significant threat by content interaction effect (*F*_(1,231)_ = 7.16, *p* = .001), indicating that higher levels of threat experiences were associated with decreased negative-neutral scene discrimination for social content but not for non-social content (Figure 2c left). With regard to neglect experiences, there was also a tendency for neglect by content interaction effect (*F*_(1,231)_ = 3.88, *p* = .05), indicating an opposite modulating effect, that is higher levels of neglect experiences were associated with decreased negative-neutral discrimination for the non-social relative to the social scenes (Figure 2c right), which did not reach significance, however. Individual variability in severity of symptomatology did not modulate this discrimination pattern (all *p* > .12). None of the other variables nor their interaction was significantly related to neural responses (all *p* > .05).

### Neural sensitivity and visual looking patterns towards negative versus neutral scenes with social and non-social content in the multi-input paradigm

**Figure 3.**
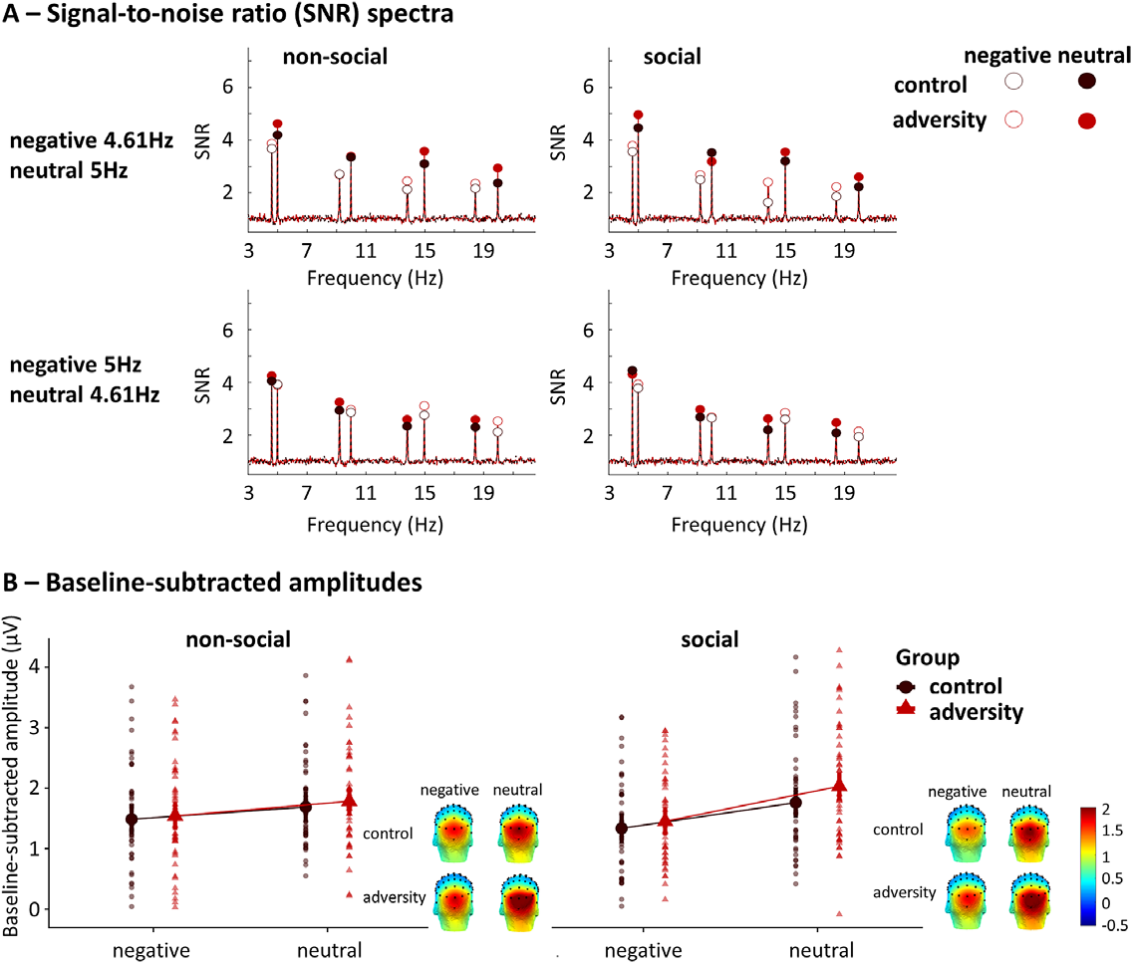
(a) SNR spectra of the neural responses at the MO region for negative and neutral scenes with social and non-social content in each group. The significant first four harmonics are displayed. (b) Baseline-subtracted amplitudes averaged across the four harmonics for negative and neutral scenes with social and non-social content in each group and the corresponding scalp distributions.

**Neural sensitivity.** Both negative and neutral scenes with social and non-social content elicited robust responses at each frequency and their harmonics in the medial occipital cortex (Figure 3a). Statistical analysis of Model_1_, contrasting both participant groups, revealed no main effect of group (*F*_(1,85)_ = 0.84, *p* = .362) nor content (*F*_(1,610)_ = 0.15, *p* = .701), a main effect of valence (*F*_(1,610)_ = 46.89, *p* < .001; that is larger neural salience towards neutral scenes than negative scenes), and valence by content interaction effect (*F*_(1,611)_ = 7.07, *p* = .008), which was driven by a larger difference between neural salience towards negative and neutral scenes with the social relative to the non-social content (*t*_(610) social negative-neutral_ = −6.77, *p* < .001; *t*_(612) nonsocial negative-neutral_ = −2.94, *p* = .018) (Figure 3b). There was also a main effect of sex (*F*_(1,85)_ = 6.05, *p* = .016), which is generally lower neural salience in males relative to females.

When entering childhood adversity as a continuous measure within the adversity group (Model_2_), we observed a main effect of valence (*F*_(1,304)_ = 29.04, *p* < .001) and a significant valence by content interaction effect (*F*_(1,305)_ = 5.14, *p* = .024), indicating higher neural salience towards neutral relative to negative scenes, but only in the social context (*t*_(306) negative-neutral_ = −5.47, *p* < .001) and not in the non-social context (*t*_(307) negative-neutral_ = −2.18, *p* = .132). No significant association was observed among individual differences in adversity or other variables on the one hand, and neural saliency of scene processing on the other (all *p* > .06). The Model_3_, disentangling the impacts of threat and neglect experiences, revealed neither dimension was associated with the neural salience of scene processing (all *p* > .053).

**Figure 4.**
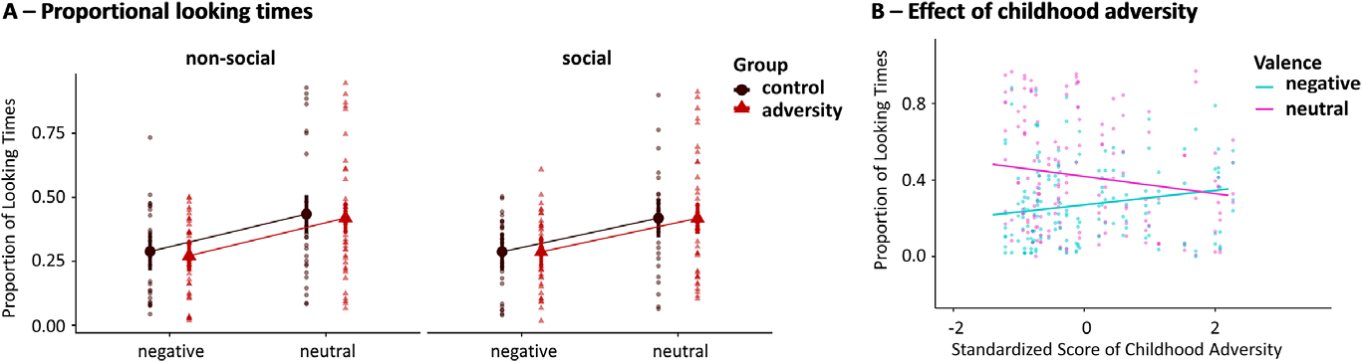
(a) The average proportion of looking times. Longer-looking times were present for neutral scenes versus negative scenes across the two groups and social and non-social content. (b) Childhood adversity was associated with increased proportional looking times towards negative scenes, although, overall, neutral scenes were still looked at more than negative scenes. Standardized scores were used.

**Visual looking patterns.** Statistical analysis of Model_1_ revealed a main effect of valence (*F*_(1,476)_ = 41.76, *p* < .001), with longer looking times to neutral scenes than to negative scenes (Figure 4a). No group- nor content-related effect was observed (all *p* > .36), indicating a general preferential looking towards neutral scenes. A more fine-grained analysis (Model_2_) in terms of individual differences in childhood adversity within the adversity group revealed again a main effect of valence (*F*_(1,271)_ = 27.90, *p* < .001), with longer looking times to neutral than to negative scenes, and significant adversity by valence interaction effect (*F*_(1,271)_ = 5.03, *p* = .026), indicating that higher levels of childhood adversity were associated with increased proportional looking times towards negative scenes, although, overall, neutral scenes were still inspected more than negative scenes (Figure 4b). No other significant effect was observed (all *p* > .28). Further disentangling the two dimensions of adversity (Model_3_) revealed no specific threat- nor neglect-related effect (all *p* > .48). As expected (Isaev et al., 2020; Vettori et al., 2020), individual differences in preferential looking times and neural saliency were highly correlated across both groups (social context: *r*_(65)_ = 0.77, *p* < .001; non-social context: *r*_(64)_ = 0.82, *p* < .001).

## Discussion

The current study investigated the processing of complex naturalistic scenes in terms of both the affective valence and the social versus non-social dimension using frequency-tagging EEG. Moreover, here, we applied and studied it in relation to childhood adversity in a young adult sample with psychiatric symptomatology. With two state-of-the-art visual paradigms, our current study objectively quantified the neural sensitivity for automatically discriminating negative from neutral scenes within a single glance, as well as the neural salience and preferential looking towards simultaneously presented negative versus neutral scenes, and this both within a social and a non-social context. Results revealed that controls showed better neural discrimination of negative from neutral scenes with social relative to non-social content, but no such distinction was observed in the adversity group. A more fine-grained analysis within the adversity group revealed that higher levels of threat experiences were associated with decreased discrimination of negative from neutral scenes, but only in a social context. When negative and neutral scenes were presented simultaneously (with either social or non-social content in different trials), we found an enhanced neural salience of neutral relative to negative scenes across both groups and across both the social and non-social content, indicating general preferential processing of neutral versus negative scenes. Simultaneously recorded eye-tracking data suggest that the enhanced neural salience of the neutral scenes may be driven by increased looking times to the neutral as compared to the negative streams across both contents and groups. Noteworthy, however, higher levels of adversity were related to relatively increased looking times towards the negative scenes versus neutral scenes.

The absence of a social content facilitatory effect (that is larger neural discrimination for social relative to non-social negative scenes from neutral scenes detected in controls) in individuals with adversity, and the association between the intensity of their threat experiences and the decrease in social threat-safety (that is negative versus neutral scenes) discrimination is informative in three ways. First, this decreased threat-safety discrimination of social information in relation to threat experiences corresponds seamlessly with our findings on facial expression processing within the same sample (Qiao et al., 2024). There, we showed a decreased neural discrimination of anger from neutral faces in relation to threat experiences, which was driven by a more negative perception of the neutral faces. Together, this suggests a general adversity-related decrease in threat-safety discrimination across a broader range of visual social stimuli, that is context-free facial expressions and complex social scenes.

Second, our findings suggest the separability of social and non-social threat discrimination in relation to adversity and the presence of a selective deficit in neural social threat discrimination in relation to threat experiences. The observed social-content-facilitated threat discrimination in controls aligns with the general social preference phenomenon in humans (Lee & Green, 2016; Vettori et al., 2020), which naturally supports the processing of emotionally arousing social information (Löw et al., 2013). The absence of such a facilitatory effect in individuals with adversity corresponds to observed alterations in social emotion processing in the context of psychopathology (Bodapati & Herbener, 2014; Lee & Green, 2016; Zivotofsky et al., 2008). For instance, while healthy controls detected threat-related targets more efficiently than non-threat targets in both a social and a non-social task, schizophrenia patients only showed such threat effect in the non-social task, implicating an impairment in social threat detection (Pinkham et al., 2014). Against this background, we may cautiously consider that the selective alteration in social threat processing in relation to childhood adversity may serve as a potential mechanism linking it to psychopathology. While no direct association between neural discrimination responses and psychiatric symptomatology was observed in our study, further studies with longitudinal follow-up and the inclusion of individuals with more severe symptoms would be informative. Childhood adversity is clearly associated with poor social functioning in individuals with psychiatric disorders (Hjelseng et al., 2022), particularly psychotic disorders (Christy et al., 2023). Possibly, the decreased social threat-safety discrimination in adversity individuals may lead to a negative perception of social situations and reduce their motivation to engage in social contact, ultimately resulting in poor social functioning. Future studies may investigate whether the distinct impact of threat experiences on social versus non-social threat-safety discrimination relates to specifically altered brain development in individuals exposed to adversity. For instance, cortical regions such as the medial prefrontal cortex have been associated with social mentalizing and the prediction of mental states of others (Atzil et al., 2023), and have consistently shown alterations in volume and thickness in individuals with threat experiences (McLaughlin, Weissman, & Bitrán, 2019).

Third, our findings provide further support for the dimensional framework of childhood adversity, and demonstrate that threat and neglect experiences may have a differential impact on the processing of emotional and threatening information (McLaughlin et al., 2014). In our study of facial expression processing (Qiao et al., 2024), we found that only threat experiences were associated with decreased angry-neutral face discrimination, and this modulating effect was not present for deprivation experiences. Our current study extends these findings by showing that more severe threat experiences were associated with decreased neural discrimination of negative versus neutral scenes, in particular scenes displaying social context. Surprisingly, however, there was also a tendency in the opposite direction with higher levels of neglect experiences being associated with reduced sensitivity to discriminate among negative and neutral non-social relative to social visual scenes, thus impacting the most on reducing sensitivity for valences in the non-social dimension. This tendency appears even more significant when not controlling for the psychiatric symptoms in our Supplementary Analysis. While this finding tends to hint towards a habituation or desensitization process for non-social negative environments in individuals with neglect experiences, empirical evidence on the impact of neglect on brain and emotion development is inconsistent (Doretto & Scivoletto, 2018; McLaughlin et al., 2019).

By presenting negative versus neutral scenes simultaneously, we found generally preferential neural processing of neutral relative to negative scenes across both contents and both groups, suggesting that both groups are equally sensitive to the emotional valence of the scenes. This contrasts with the findings of our face processing study where we observed that the adversity group displayed a relatively enhanced neural preference for angry faces as compared to controls, as well as a more indistinct processing of angry versus neutral faces (Qiao et al., 2024). A potential explanation is that adversity-related enhanced neural salience toward threatening information might be limited to certain facial expressions such as angry/fearful faces (Dannlowski et al., 2012), while the images of the scenes in our current study varied widely. Yet, simultaneously recorded eye-tracking data did reveal that, although neutral scenes were generally looked at more compared to negative scenes, higher levels of adversity exposure were related to increased looking times towards negative relative to neutral scenes. This again suggests a relative indifference towards the emotional valence (that is negative versus neutral) of the scenes in individuals with adversity. Thus, taken together, across both studies and both types of stimulus material, there is converging evidence that individuals exposed to early adversity show a reduced neural attentional discrimination of threatening facial information and a reduced behavioral attentional discrimination of threatening scene information.

A limitation of our study is that our measures of childhood adversity rely on retrospective self-reports, which necessarily entail a possibility of recall bias (Newbury et al., 2018; Reuben et al., 2016). Furthermore, we only included emotional and physical neglect in the deprivation dimension, thus our results do not generalize to other deprivation categories such as poverty. In addition, a sensitive period of adversity exposure has been suggested (Gabard-Durnam & McLaughlin, 2019; Teicher, Samson, Anderson, & Ohashi, 2016) and a more comprehensive assessment comprising the age of exposure may further clarify the association among childhood adversity, emotion processing, and psychopathology. Second, while the sex ratio of our sample is representative of adversity populations (Giano, Wheeler, & Hubach, 2020; Knipscheer et al., 2020), that is females have greater exposure to adverse events than males, the restricted involvement of male participants may limit the generalizability of our findings and impede formal gender comparisons. Also, there is an age difference between the control and the adversity group in the present study. However, no age-related effect was observed and all analyses controlled for the possible effects of age. Future studies involving different age groups would be informative in clarifying the potential developmental effects. Moreover, here, we only include images of negative and neutral social or non-social scenes, thus, it remains uncertain whether childhood adversity may also impact the processing of positively valued visual scenes.

In conclusion, the current study introduced the frequency-tagging EEG approach to investigate the neural and attentional processing of complex social and non-social visual scenes in young adults with a history of childhood adversity. The absence of a social content facilitatory effect on threat-safety discrimination in the adversity group suggests a selective alteration in social information processing relating to early adverse experiences. Furthermore, the association of higher levels of threat experiences with decreased neural discrimination of negative versus neutral social scenes corresponds to our findings on facial expression processing in this same population, indicating adversity-related (and more specifically threat-related) deficits in automatic threat-safety discrimination across a broader range of social visual stimuli. This decreased threat-safety discrimination in young adults exposed to early adversity might impact individuals’ perception of real-life social situations and may result in poor social functioning and enhanced social stress.

## Supporting information

Qiao et al. supplementary material Qiao et al. supplementary material

## Supplementary material

The supplementary material for this article can be found at http://doi.org/10.1017/S0033291725000029.
