# Supplementary material for: Implicit neural sensitivity for negatively valued social and non-social visual scenes in young adults exposed to childhood adversity
Source: Psychol Med. 2025 Feb 11;55:e37. doi: 10.1017/S0033291725000029 (PMC12017353; doi:10.1017/S0033291725000029)
Supplement: Qiao et al. supplementary material [file S0033291725000029sup001.docx]

**Implicit neural sensitivity for negatively valued social and non-social visual scenes in young adults exposed to childhood adversity**

***Supplemental Information***

# **Supplementary Methods**

*Participants*

The exclusion criteria included: being actively in psychotropic treatment, medical or neurological disorders impeding participation (i.e. refractory diabetes, disabling heart conditions, refractory epilepsy or neuromuscular diseases), current use (or use in the month preceding the study) of psychoactive substances, alcohol consumption >14 units for both men and women according to the NICE guidelines on harmful use (NICE Guidelines, 2010), active suicidality and autism spectrum disorder.

Before inclusion, all participants and -when below 18 years- one of their legal guardians signed an informed consent conform to the new General Data Protection Regulations (GDPR) established by the European Parliament.

*Measures*

**Childhood adversity.** Exposure to adverse experiences before the age of eighteen was measured using a modified screening version of the Juvenile Victimization Questionnaire 2nd revision (Adult Retrospective Form; JVQ-R2) (Finkelhor, Hamby, Ormrod, & Turner, 2005) and 5 questions of the Emotional Neglect subscale of the Childhood Trauma Questionnaire (CTQ) (Bernstein & Fink, 1998). A total of 33 items were included, covering 7 categories of childhood adversity (peer and sibling victimization/bullying, physical abuse, physical neglect, emotional abuse, emotional neglect, sexual abuse and domestic violence). For each item, participants reported the exposure (yes or no) and, if yes, the frequency (1=once, 2=seldom, 3=sometimes, 4=often, 5=very often) of that experience. They were included in the adversity group if they answered yes, with a frequency of at least “sometimes”, (i) to one of the items in the categories peer and sibling victimization, emotional neglect and domestic violence, or (ii) to the item of the emotional abuse category (“At any time in your life, did you get scared or feel really bad because grown-ups in your life called you names, said mean things to you, or said they didn’t want you?”), or (iii) to one of the two items of the sexual abuse category (“Did anyone ever force you to look at his or her intimate parts by forcing or surprising you or by suddenly showing you?”, “Did anybody ever hurt your feelings by saying or writing something sexual about you or your body?”). They were also included in the adversity group if they answered yes, with any frequency, to one of the items in the categories physical abuse, physical neglect, emotional abuse (except for the item above) and sexual abuse (except for the two items above). These cut-off scores were made following the rationale of Croft and colleagues (Croft et al., 2019). During the testing session, exposure to childhood adversity was measured again to assess individual differences in adversity levels (i.e. adversity exposure being quantified as a continuous score). Specifically, for general adversity exposure (i.e. regardless of the dimensions), we created a continuous score by averaging the mean frequency (i.e. mean score across items for each category) of the seven adversity categories. Likewise, to obtain an index of neglect and threat experiences, we averaged the mean frequency of physical and emotional neglect and the mean frequency of the other five adversity categories, respectively. These scores were created for dimensional analyses to investigate how individual differences in adversity impact the responses (see Methods).

**Symptoms.** Depressive symptoms were measured using the Beck Depression Inventory (BDI-II), with a predefined cut-off score equal to or more than 11 to indicate the presence of depression (Beck, Steer, & Brown, 1996; Whisman & Richardson, 2015). Anxiety was measured using the trait scale of the State Trait Anxiety Inventory, with a cut-off score equal to or more than 40 indicating the display of anxiety (Addolorato et al., 1999; Dennis, Coghlan, & Vigod, 2013; Spielberger, Gorsuch, Lushene, Vagg, & Jacobs, 1983). Psychotic symptoms were measured using the Prodromal Questionnaire-16 (PQ-16), with a cut-off score equal to or more than 5 on the symptom scale or equal to or more than 8 on the distress scale to indicate the display of psychotic symptoms (Savill, D’Ambrosio, Cannon, & Loewy, 2018). During the testing session, symptoms were measured again using the same questionnaire and the scores were used for the dimension analyses (see Methods). All measures were conducted in REDCap (<https://www.project-redcap.org/>).

*Stimuli and Procedure*

**Stimuli.** Stimuli were selected from the Nencki Affective Picture System (Marchewka, Żurawski, Jednoróg, & Grabowska, 2014) and comprised sets of social negative, social neutral, non-social negative and non-social neutral scenes, each with 25 images. Social scenes were selected from the “people” and “face” categories; non-social scenes were selected from the “animal”, “object” and “landscape” categories. Based on the normative ratings of each image of the database (ranging from 1 “*very negative*” to 9 “*very positive*”, with 5= *neutral*), we selected those images depicting negative scenes (i.e. all rated below 3.32 on the valence parameter) and neutral scenes (i.e. rated between 4.40 and 5.91). Negative scenes differed highly significantly from neutral scenes in terms of valence and arousal for both the social (valence: *t*_(48)_ = -23.40, *p* < .001; arousal: *t*_(48)_ = 21.85, *p* < .001) and non-social category (valence: *t*_(48)_ = -24.16, *p* < .001; arousal: *t*_(48)_ = 17.94, *p* < .001), while there was no difference between the social and non-social negative scenes (valence: *t*_(48)_ = 1.22, *p* = .227; arousal: *t*_(48)_ = -1.64, *p* = .106) and neutral scenes (valence: *t*_(48)_ = -0.62, *p* = .538; arousal: *t*_(48)_ = -0.87, *p* =.387). Additionally, based on the information regarding physical stimulus properties provided by Nencki Affective Picture System, the selected four categories of images were balanced in terms of luminance (*F*_(3,96)_ = 0.57, *p* = .634), contrast (*F*_(3,96)_ = 0.77, *p* = 0.516) and complexity (JPEG size: *F*_(3,96)_ = 0.52; *p* = 0.673; entropy: *F*_(3,96)_ = 1.01; *p* = 0.390). All stimuli were set to a size of 320 × 240 pixels and presented on an LCD 24-in computer screen with a resolution of 2560 × 1440 and a refresh rate of 60 Hz. Participants were seated in a dimly lit room at 60 cm viewing distance of the screen and were instructed to maintain a constant distance while recording the data.

**The oddball frequency-tagging EEG paradigm.** As illustrated in figure 1A, stimuli were presented on the screen through sinusoidal contrast modulation on a light grey background. Specifically, a series of base stimuli (i.e., neutral scenes) were displayed at 5 Hz, periodically interleaved with a negative scene every fourth image (i.e., oddball stimuli displayed at 5 Hz/4 = 1.25 Hz). Each sequence lasted 60 s flanked by 2 s of fade-in and fade-out, with the stimulus contrast gradually increasing (0–100%) or decreasing (100–0%), respectively. In total, four social and four non-social sequences were included, and the order was randomized for each participant. All images of scenes were drawn randomly from their respective categories, cycling through all available images before any image repetition. To ensure attentiveness of the participants, an orthogonal task was implemented. Participants had to press a key whenever they detected a brief (300 ms) color change (black to red, occurring randomly 12 times within each sequence) of the fixation cross presented in the center of the images.

**The multi-input frequency-tagging EEG paradigm.** This paradigm consisted of two simultaneously presented streams of negative and neutral scenes, each labelled at a different presentation rate (4.61Hz vs. 5Hz or vice versa) and displayed at either the left or right visual filed. The presentation rate and the position on the screen were counterbalanced for the two categories of emotional scenes. In total, four social and four non-social sequences were included, and the order was randomized for each participant. Each sequence lasted 30 s, flanked by 2 s of interval at the beginning and the end. Stimuli were presented with a square wave, i.e., reaching full contrast (0–100%) within one frame of the screen refresh rate. All images of scenes were drawn randomly from their respective categories, cycling through all available images before any image repetition. A similar orthogonal task was administered: participants had to press a key as soon as they noticed a briefly (300 ms) appeared rectangle outside the two images of scenes (occurring randomly 8 times within each sequence). The order of the two paradigms was randomized across participants.

**EEG data recording.** We recorded the EEG signal using a BioSemi Active-Two amplifier system with 64 Ag/AgCl electrodes and two additional electrodes (common mode sense and driven right leg) as reference and ground electrodes. Horizontal eye movements were recorded via two external electrodes placed at the outer canthi of both eyes and vertical eye movements were captured via one external electrode above and one below the right eye. The data were sampled at 512 Hz.

**Eye-tracking data recording.** For the multi-input paradigm, simultaneous eye-tracking data were recorded using a Tobii X3-120 screen-based remote eye-tracker and Tobii Pro software (Tobii Pro) with a sample rate of 120 Hz. In addition to the standard calibration procedure of the Tobii X3-120 (i.e. participants had to follow a white dot appearing at five locations across the screen), a calibration validation procedure (Vettori et al., 2020) was administered. Participants were instructed to fixate at the center of ten consecutive fixation crosses appearing at different locations across the screen. This procedure allowed us to obtain a subject-specific quantitative measure of data quality, including an index of error angle (mean and variance) and the resulting accuracy, by calculating the angle between the vectors to the displayed fixation cross versus the actual gaze point. These values were used when attributing gaze points to particular areas of interest (AOIs).

*Data analysis*

**EEG data.** Data preprocessing was conducted using Letswave 6 (https://www.letswave.org/) and MATLAB 2021 (MathWorks). Specifically, the raw EEG signal was cropped into segments with 4 s before and 6 s after each sequence: 70-s segments for the oddball paradigm and 40-s segments for the multi-input paradigm. Thereafter, the EEG data were bandpass filtered (0.1 to 100 Hz) using a fourth-order Butterworth filter and down sampled from 512 Hz to 256 Hz. Noisy channels were linearly interpolated using the three spatially nearest electrodes (not more than 5% of the electrodes, i.e. three electrodes, were interpolated). All data segments were re-referenced to a common average reference. The preprocessed segments of the oddball paradigm were further cropped to contain an integer number of 1.25 Hz cycles, starting from the beginning of the sequence until 59.332 s (15,189 time bins). The resulting segments in the time domain were averaged for each stimulus type for each participant and transformed into the frequency domain with a Fast Fourier transform (FFT), yielding a spectrum between 0 and 127.9747 Hz. For the multi-input paradigm, the preprocessed segments were cropped to contain an integer number of 0.39 Hz cycles (i.e. the difference between 4.61 and 5 Hz), starting from the beginning of the sequence until 28.2695 s (7,237 time bins). The resulting segments in the time domain were averaged per presentation rate for each stimulus type for each participant and transformed into the frequency domain with FFT, yielding a spectrum between 0 and 127.9469 Hz.

**
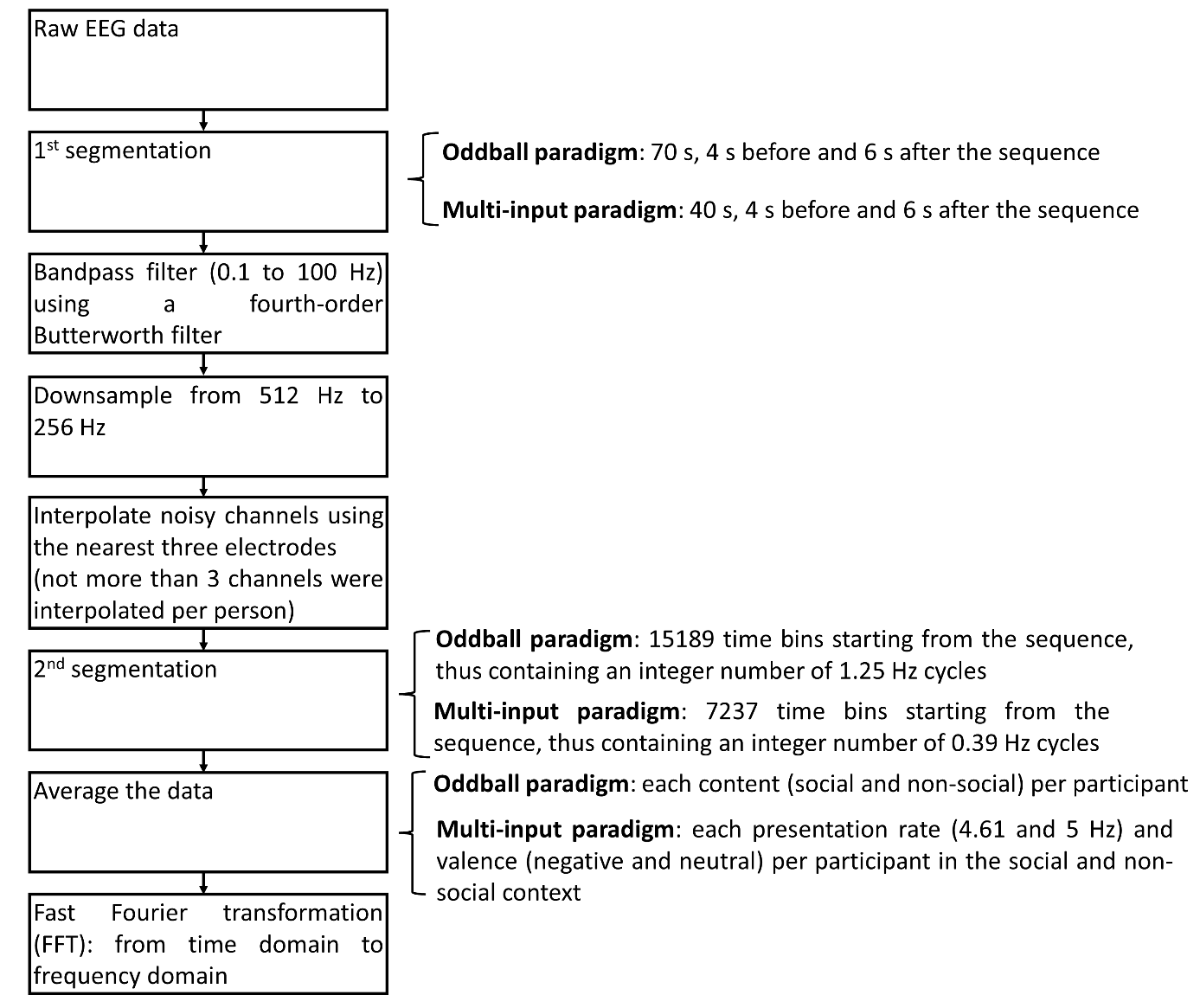
**

**Figure S1.** The flowchart of EEG data preprocessing.

**Eye-tracking data.** In line with a previous study (Vettori et al., 2020), we analyzed eye-tracking data using a series of custom-built scripts in Matlab (The Mathworks; https://github.com/TimVanWesemael/Fuzzy-AOI-EyeTracking). Raw data were filtered using the I2MC algorithm (Hessels, Niehorster, Kemner, & Hooge, 2017) to delete random noise, interpolate missing data and identify fixations. Two areas of interest (AOIs; left and right visual region) were defined as the rectangular areas (320 × 240 pixels) where the two streams of images were presented. An ‘outside AOI’ was also defined to label all the fixation points that were not attributed to the two scene AOIs.

We used a probability weighting approach to allocate fixations to the AOIs, while taking the subject-specific data quality into account. For the specific methods, please refer to previous studies (Qiao et al., 2024; Van der Donck et al., 2021; Vettori et al., 2020). Thereafter, the relative duration of all fixation points on each AOI was averaged over the two trials (i.e., negative scenes presented at either the left or right visual region in the social and non-social context, respectively). Proportional looking times for the two scene AOIs and the ‘outside AOI’ were calculated.

**Orthogonal task performance.** Both accuracy and reaction times were calculated to evaluate the performance. A key press was considered correct if it occurred in the 100 to 2000 ms time window following an actual color change. For both the oddball and the multi-input paradigm, the proportions and reaction times of the correct responses were averaged across the four sequences, resulting in an overall accuracy and reaction times per content condition (i.e. social and non-social) per participant.

*Statistical analysis*

**Orthogonal task performance.** Similar to the main analysis, linear mixed models (LMM) using *R*-package *afex*, version 1.2.1 (Singmann, Bolker, Westfall, Aust, & Ben- Shachar, 2020), were performed. We fitted a separate model for accuracy and reaction times, with Content [social and non-social] and Group [control and adversity] as the fixed factors for both the oddball and multi-input paradigm. A random intercept by subject was included to account for the clustered nature of the repeated measurements. Age and sex were added into the models as covariates.

**Exploratory analyses.** We constructed additional models to explore the effect of individual variability in severity of adversity and symptomatology (i.e. as continuous measures), while not controlling for each other, within the adversity group. Specifically, for the neural discriminative responses in the oddball paradigm, we constructed the following five models: a Model_1_ *y ~ age + sex + Childhood adversity * Content + (1|subject)*, a Model_2_ *y ~ age + sex + Threat * Content + Neglect * Content + (1|subject), a Model_3_ y ~ age + sex + Depression * Content + (1|subject)*, a Model_4_ *y ~ age + sex + Anxiety * Content + (1|subject)*, and a Model_5_ *y ~ age + sex + Psychosis * Content + (1|subject)*, with Content [social vs. non-social] as the within-subjects effect. For the neural sensitivity and visual looking patterns measured in the multi-input paradigm, five similar models were fitted, with Content [social vs. non-social] and Valence [negative vs. neutral] as the within-subject effects. Continuous scores of childhood adversity and the two dimensions and symptoms during testing were used and all scores were standardized to have a mean of 0 and a standard deviation of 1 before being added into the models.

# **Supplementary Results**

*Demographic information*

Data of measures were downloaded from REDCap, version “ENGAGE_DATA_2022-11-06_1208”. Based on the sample included in the oddball and multi-input paradigm (43 controls and 48 participants with adversity), we found excellent test-retest reliability in the adversity group for childhood adversity [*r*_(46)_ = .83, *p* < .001], threat experiences [*r*_(46)_ = .80, *p* < .001] and neglect experiences [*r*_(46)_ = .77, *p* < .001], as well as in both groups for depression [adversity group: *r*_(46)_ = .80, *p* < .001; control group: *r*_(41)_ = .59, *p* < .001)], anxiety [adversity group: *r*_(46)_ = .70, *p* < .001; control group: *r*_(41)_ =.66, *p* < .001] and psychotic [adversity group: *r*_(46)_ = .83, *p* < .001; control group: *r*_(41)_ = .83, *p* < .001] symptoms.

We further checked the association between childhood adversity and symptoms. Results revealed that individual differences in childhood adversity were associated with the presence of depressive (*r*_(46)_ = .44, *p* = .002), anxiety (*r*_(46)_ = .46, *p* = .001) and psychotic (*r*_(46)_ = .44, *p* = .002) symptoms. When disentangling the two dimensions, we found that threat was associated with depressive (*r*_(46)_ = .50, *p* < .001), anxiety (*r*_(46)_ = .51, *p* < .001) and psychotic (*r*_(46)_ = .48, *p* < .010) symptoms. No association was detected between neglect and any of the symptoms.

*The oddball paradigm*

**Orthogonal task performance.** For the reaction times, results showed equal performances for the two groups (*M*_control_ = 0.47s, *SD*_contro_l = 0.05; *M*_adversity_ = 0.47s, *SD*_adversity_ = 0.05), *F*_(1,85)_ = 0.07, *p* = .794, and in the two social contexts (*M*_social_ = 0.47s, *SD*_social_ = 0.05; *M*_non-social_ = 0.46s, *SD*_non-social_ = 0.05), *F*_(1,87)_ = 3.78, *p* = .055. For the accuracy, results also showed equal performance for the two groups (*M*_control_ = 97.9%, *SD*_control_ = 3.77%; *M*_adversity_ = 98.1%, *SD*_adversity_ = 3.98%), *F*_(1,85)_ = 0.07, *p* = .790, and in the two social contexts (*M*_social_ = 98.0%, *SD*_social_ = 3.58%; *M*_non-social_ = 98.0%, *SD*_non-social_ = 4.16%), *F*_(1,87)_ < 0.01, *p* = .989.

**Neural responses to the visual base stimulation.** Neural responses to the visual base stimulation (i.e. 5 Hz) were quantified as the summed responses of four harmonics, i.e., 5, 10, 15 and 20 Hz. A linear mixed model (*y ~ age + sex + Group * Content * ROI + (1|subject)*) was constructed and revealed a main effect of ROI (*F*_(2,437)_ = 197.24, *p* < .001): lower responses in the LOT region relative to the ROT region (*t*_(437)_ = -8.27, *p* < .001) and in both regions relative to the MO region (LOT vs. MO: *t*_(437)_ = -19.79; ROT vs. MO: *t*_(438)_ = -11.60, both *p* < .001). There was also a main effect of sex, with lower responses in male participants relative to female participants, *F*_(1,87)_ = 4.62, *p* = .034. The absence of any other significant main and/or interaction effect indicates a similar synchronization to the flickering stimuli in both groups and across both the social and non-social condition.

**Exploratory analyses of associations with the neural discriminative responses.** Exploratory analyses were conducted to investigate the impact of adversity and symptomatology as continuous measures, while not controlling for each other, within the adversity group. Model_1_, exploring the impact of general adversity exposure, revealed neither a main effect of childhood adversity (*F*_(1,44)_ = 1.29, *p* = .262) nor a childhood adversity by content interaction effect (*F*_(1,235)_ = 0.186 *p* = .667). Model_2_, disentangling the impact of threat and neglect experiences, showed a significant threat by content interaction effect (*F*_(1,234)_ = 5.03, *p* = .026), indicating that more frequent and more severe early threat experiences were related to a decreased neural discrimination of negative versus neutral scenes with a social content relative to a non-social content. This is consistent with our findings in the main analyses after controlling for individual differences in symptom load, emphasizing the robustness of the modulating effect of threat experiences on the neural discrimination responses. There is also a modulating effect of neglect experiences on this neural discrimination response if we don’t control for any of the other variables (*F*_(1,234)_ = 3.95, *p* = .048), i.e. more frequent and more severe early neglect experiences were related to a decreased neural discrimination of negative versus neutral scenes with a non-social content relative to a social content. However, this interaction effect is no longer significant after controlling for current symptoms, as reported in the main manuscript. Exploratory analyses of the impact of symptomatology on implicit neural discrimination (Model_3_, Model_4_ and Model_5_ for depressive, anxiety and psychotic symptoms, separately), while not controlling for adversity, showed no significantly modulating effect of neither symptom (all *p* > .31), further confirming our findings in the main text that individual variability in severity of symptomatology did not modulate the neural sensitivity for scene processing in our participant samples.

*The multi-input paradigm*

**Orthogonal task performance.** For the reaction times, results also revealed equal performances for the two groups (*M*_control_ = 0.44s; *M*_adversity_ = 0.45s), %), *F*_(1,87)_ = 0.25, *p* = .618, and under the two social conditions (*M*_social_ = 0.44s; *M*_non-social_ = 0.45s), *F*_(1,89)_ = 1.12, *p* = .294. For the accuracy, results also showed equal performances for the groups (*M*_control_ = 95.8%; *M*_adversity_ = 94.1%), *F*_(1,87)_ = 1.03, *p* = .312, and under the two social conditions (*M*_social_ = 95.5%; *M*_non-social_ = 94.3%), *F*_(1,89)_ = 2.15, *p* = .146.

**Exploratory analyses of associations with the neural responses.** Model_1_, investigating the effect of individual differences in childhood adversity while not controlling for symptoms, revealed no modulating effect of adversity exposure. Model_2_, investigating the effect of threat and neglect experiences while not controlling for symptoms, revealed a significant valence by content by threat interaction effect (*F*_(1,310)_ = 5.28, *p* = .022). However, no pairwise contrast remained significant after correcting for multiple comparisons during post hoc analyses (all *p* > .06). Exploratory analyses investigating the effect of individual differences in severity of symptomatology (Model_3_, Model_4_ and Model_5_ for depressive, anxiety and psychotic symptoms, respectively) revealed no modulating effect of any of the symptoms (all *p* > .08).

**Data quality of eye-tracking.** T-tests of the data quality obtained via the calibration validation procedure demonstrated no group differences: both groups showed similar average error angles (*M*_adversity_ =0.01˚ ± 0.003˚; *M*_control_ = 0.01˚ ±0.003˚; *t*_(67)_ = 0.43, *p* = .670) and the root-mean-square of the angles (*M*_control_ = 0.01 ± 0.003; *M*_adversity_ = 0.01 ± 0.005; *t*_(67)_ = -0.12, *p* = .905).

**Exploratory analyses of associations with the visual looking patterns.** Exploratory analyses of the effect of general adversity (Model_1_), while not controlling for symptoms, on the looking times within the adversity group revealed a main effect of valence (*F*_(1,280)_ = 28.54, *p* < .001), with longer looking times to neutral scenes than to negative scenes, and a significant adversity by valence effect (*F*_(1,280)_ = 9.03, *p* = .003), indicating that higher levels of adverse experiences were associated with increased proportional looking times towards negative scenes relative to neutral scenes, though neutral scenes were generally looked at more than negative scenes. This is consistent with our findings in the main text after controlling for symptoms. Further disentangling the two dimensions of adversity (Model_2_) revealed no specific threat- nor neglect-related modulating effect (all *p* > .12). Exploratory analyses of symptom severity, while not controlling for adversity, revealed a significant depression by valence interaction effect (Model_3_, *F*_(1,280)_ = 4.47, *p* = .035), indicating that the presence and severity of depressive symptoms was associated with increased proportional looking times towards negative scenes relative to neutral scenes. This modulating effect, however, did not remain while controlling for adversity as shown in the main text. No modulating effect of anxiety (Model_4_) or psychotic (Model_5_) symptoms was observed (all *p* > .052).

# **References**

Addolorato, G., Ancona, C., Capristo, E., Graziosetto, R., Di Rienzo, L., Maurizi, M., & Gasbarrini, G. (1999). State and trait anxiety in women affected by allergic and vasomotor rhinitis. *Journal of Psychosomatic Research*, *46*(3), 283–289. doi: 10.1016/S0022-3999(98)00109-3

Beck, A. T., Steer, R. A., & Brown, G. K. (1996). *Manual for the Beck Depression Inventory-II.* San Antonio, TX: Psychological Corporation.

Bernstein, D. P., & Fink, L. (1998). *Childhood Trauma Questionnaire: A retrospective self-report manual*. San Antonio: TX: The Psychological Corporation.

Croft, J., Heron, J., Teufel, C., Cannon, M., Wolke, D., Thompson, A., … Zammit, S. (2019). Association of Trauma Type, Age of Exposure, and Frequency in Childhood and Adolescence with Psychotic Experiences in Early Adulthood. *JAMA Psychiatry*, *76*(1), 79–86. doi: 10.1001/jamapsychiatry.2018.3155

Dennis, C. L., Coghlan, M., & Vigod, S. (2013). Can we identify mothers at-risk for postpartum anxiety in the immediate postpartum period using the State-Trait Anxiety Inventory? *Journal of Affective Disorders*, *150*(3), 1217–1220. doi: 10.1016/j.jad.2013.05.049

Finkelhor, D., Hamby, S. L., Ormrod, R., & Turner, H. (2005). The Juvenile Victimization Questionnaire: Reliability, validity, and national norms. *Child Abuse & Neglect*, *29*, 383–412. doi: 10.1016/j.chiabu.2004.11.001

Hessels, R. S., Niehorster, D. C., Kemner, C., & Hooge, I. T. C. (2017). Noise-robust fixation detection in eye movement data: Identification by two-means clustering (I2MC). *Behavior Research Methods*, *49*, 1802–1823. doi: 10.3758/s13428-016-0822-1

Marchewka, A., Żurawski, Ł., Jednoróg, K., & Grabowska, A. (2014). The Nencki Affective Picture System (NAPS): Introduction to a novel, standardized, wide-range, high-quality, realistic picture database. *Behavior Research Methods*, *46*, 596–610. doi: 10.3758/s13428-013-0379-1

NICE Guidelines. (2010). *Alcohol-use disorders: prevention*.

Qiao, Z., Samaey, C., Donck, S. Van de, Jennen, L., Mazereel, V., Winkel, R. van, & Boets, B. (2024). Early Experience of Threat is Associated with Altered Neural Sensitivity for Facial Expressions in Young Adults with Emerging Symptoms. *PsyArXiv Preprint*. doi: 10.31234/osf.io/qf6e2

Savill, M., D’Ambrosio, J., Cannon, T. D., & Loewy, R. L. (2018). Psychosis risk screening in different populations using the Prodromal Questionnaire: A systematic review. *Early Intervention in Psychiatry*, *12*, 3–14. doi: 10.1111/eip.12446

Singmann, H., Bolker, B., Westfall, J., Aust, F., & Ben- Shachar, M. S. (2020). *Package ‘afex.’*

Spielberger, C. D., Gorsuch, R. L., Lushene, R., Vagg, P. R., & Jacobs, G. A. (1983). *State-Trait Anxiety Inventory for Adults*. Consulting Psychologists Press.

Van der Donck, S., Vettori, S., Dzhelyova, M., Mahdi, S. S., Claes, P., Steyaert, J., & Boets, B. (2021). Investigating automatic emotion processing in boys with autism via eye tracking and facial mimicry recordings. *Autism Research*, *14*, 1404–1420. doi: 10.1002/aur.2490

Vettori, S., Dzhelyova, M., Van der Donck, S., Jacques, C., Van Wesemael, T., Steyaert, J., … Boets, B. (2020). Combined frequency-tagging EEG and eye tracking reveal reduced social bias in boys with autism spectrum disorder. *Cortex*, *125*, 135–148. doi: 10.1016/j.cortex.2019.12.013

Whisman, M. A., & Richardson, E. D. (2015). Normative Data on the Beck Depression Inventory - Second Edition (BDI-II) in College Students. *Journal of Clinical Psychology*, *71*(9), 898–907. doi: 10.1002/jclp.22188
